# Burnout, work engagement and work hours – how physicians’ decision to work less is associated with work-related factors

**DOI:** 10.1186/s12913-023-09161-9

**Published:** 2023-02-15

**Authors:** FU Jung, E Bodendieck, M Bleckwenn, FS Hussenoeder, M Luppa, SG Riedel-Heller

**Affiliations:** 1 Institute of Social Medicine, Occupational Medicine and Public Health, Medical Faculty, Leipzig University, Ph.-Rosenthal-Str. 55, 04103 Leipzig, Germany; 2 General Practice, Dresdner Straße 34a, 04808 Wurzen, Germany; 3 Department of General Practice, Medical Faculty, Leipzig University, Ph.-Rosenthal-Str. 55, 04103 Leipzig, Germany

**Keywords:** Primary care, General practice, Burnout, Work hour, Work engagement, Occupational health

## Abstract

**Background:**

According to new estimates, the health care sector will suffer a shortage of physicians in primary and specialty care. In this context, work engagement and burnout are two constructs that have gained attention recently. The aim of this study was to investigate how these constructs are related to work hour preference.

**Method:**

The present study was based on the baseline survey of the long-term study of physicians with different specialties, in which 1,001 physicians took part (response rate: 33.4%). Burnout was measured using the Copenhagen Burnout Inventory adapted for health care professionals; work engagement was assessed using the Utrecht Work Engagement scale. Data analyses included regression and mediation models.

**Results:**

Overall, 297 out of 725 physicians were planning to cut down work hours. Several reasons - such as burnout - are discussed. According to multiple regression analyses desire to work less hours was significantly linked to all three dimensions of burnout (p < 0.001), as well as work engagement (p = 0.001). In addition, work engagement significantly mediated the relationship between the burnout dimensions on work hour reduction (patient-related: b = − 0.135, p < 0.001; work-related: b = − 0.190, p < 0.001; personal: b = − 0.133, p < 0.001 ).

**Discussion:**

Physicians tending to reduce work hours exhibited different levels of work engagement as well as burnout (personal, patient- and work-related). Moreover, work engagement influenced the relationship between burnout and work hour reduction. Therefore, interventions that increase work engagement may positively impact negative effects of burnout on work hour changes.

## Introduction

Currently, the health care system is facing challenging times. The presence of pandemics, such as the COVID-19-pandemic crisis, as well as personal difficulties (i.e. lack of physicians) are threatening public health care by increasing distress and burnout in health care professionals. Previously, several studies suggest that burnout is common among physicians working in outpatient and inpatient care [1–3]. In the context of health care work, burnout is characterized by three different dimensions: personal, work-related and client-(or patient-) burnout [4, 5]. According to Kristensen et al. [4], personal burnout is defined as “[…] *the degree of physical and psychological fatigue and exhaustion experienced by the person”* (page 197). Work-related burnout may be understood as *“the degree of physical and psychological fatigue and exhaustion that is perceived by the person as related to his/her work“* (page 197), whereas client-related burnout implies „*the degree of physical and psychological fatigue and exhaustion that is perceived by the person as related to his/her work with clients*“ (page 197). Burnout has often been associated with high emotional load, making health care professionals and physicians especially vulnerable[4, 6]. Furthermore, it has been shown to influence physician health by increasing depressive symptoms or chronic disease [7], but also effecting quality of care, patient satisfaction and safety [8–11]. Moreover, physician burnout has been linked to physician turnover, the decision to leave the health care system or retirement plans [12–14].

One aspect linked to the high prevalence of physician burnout is the amount of work hours, especially overtime hours. Physicians’ high workload has been linked to overtime hours, the actual stress during on-call duty, the ability to take breaks, and work on weekends, during free time, and on vacation [15, 16]. In Germany, for example, working hours often exceed legal requirements and restrictions [17]. Due to recent changes in legal regularities, physicians should not be working more than 48 h per week and no longer than 12 h per day. In addition, due to the introduction of the concept of “New Work” [18], which suggests more flexibility for instance with regard to working hours, a greater number of health care professionals plan to reduce working hours in order to decrease overall burnout, leaving more time for recovery, educational purpose, or other responsibilities (i.e. care work). In this context, a long-term study investigating the relationship between burnout and professional effort in physicians (as defined by full-time equivalent (FTE) units) found that burnout may result in a reduction of work hours [18], however no causalities could be made as they did not reveal the reasons for reductions in work effort (voluntary or involuntary). Other studies suggest that working part time or generally less hours per week may decrease burnout (by increasing time for recovery) or increase burnout and workload (for the rest of the staff) by decreasing the work force without decreasing the amount of work[19, 20]. The question remains whether personal resources may be related to workload or burnout risk.

In this context, high levels of burnout among physicians have been linked to lower levels of engagement and commitment [21, 22]. On the other hand, a variety of studies suggest that work engagement may positively influence the consequences of burnout or be protective of it [23–25]. Work engagement has been described as the antithesis of burnout and refers to the relationship with ones work [26]. By definition, “*engagement is a positive, fulfilling, work-related state of mind that is characterized by vigor, dedication, and absorption. Rather than a momentary and specific state, engagement refers to a more persistent and pervasive affective-cognitive state that is not focused on any particular object, event, individual, or behavior*” [27]. Engagement has been linked to positive outcomes within the healthcare setting, including fewer medical errors, counterbalancing for job-related stress. Research concluded that while burnout was associated with self-perceived poorer patient care, work engagement on the other hand was linked to self-reported better care [28]. According to the job demands-resources model, job demands may lead to burnout as well as high adverse events (such as errors at work or accidents), whereas job resources are linked to work engagement, predicting lower adverse events [29]. The question remains how this can be applied to physicians in the context of burnout, work engagement and intention to cut down work hours, especially as work engagement has been shown to decrease turnover intention by mediating the relationship between jobs characteristics and turnover in health care professionals and physicians [30–32]. Literature concerning the relationship between burnout and work engagement, as well as possible consequences for patient care are extremely limited. So far, there are no studies that investigated possible relationships between burnout, work engagement and work hour preferences.

Therefore, the aim of this study was to investigate reasons and desire for reducing work hours and how this may be related to levels of burnout as well as work engagement exhibited by physicians. According to our hypotheses, higher burnout – being a long-term stress reaction - may be associated with the desire to reduce working hours whereas a greater amount of work engagement may result in less desire for work hour reduction. In addition, it was investigated, whether work engagement would mediate the relationship between burnout and the desire to work less.

## Method

### Study Design and Sampling

The present study was based on the baseline survey of the long-term study of doctors working in the Federal State of Saxony (Germany). The sampling and the (postal) dispatch of the questionnaires took place in February 2020. Participants were randomly selected, contacted via mail and asked to return the filled-in questionnaire. The data was pseudonymized for longitudinal use. Participants were asked to generate a personal code that consists of letters of their parents’ names and their month of birth (numerical). The procedure will be repeated in the follow-up studies (four and eight years later), allowing for anonymous matching of cases and longitudinal comparison. Overall, 1,001 physicians took part in this study by returning the questionnaire (response rate: 33.4%). For the purpose of this study, the following inclusion criteria were applied: not having reached the age of 67 (starting age of regular pension for physicians in Germany) and currently working in direct patient care (either in hospital or in an outpatient setting, employed or self-employed). The remaining sample that was used for analysis included 725 physicians.

### Assessment

**Fig. 1:**
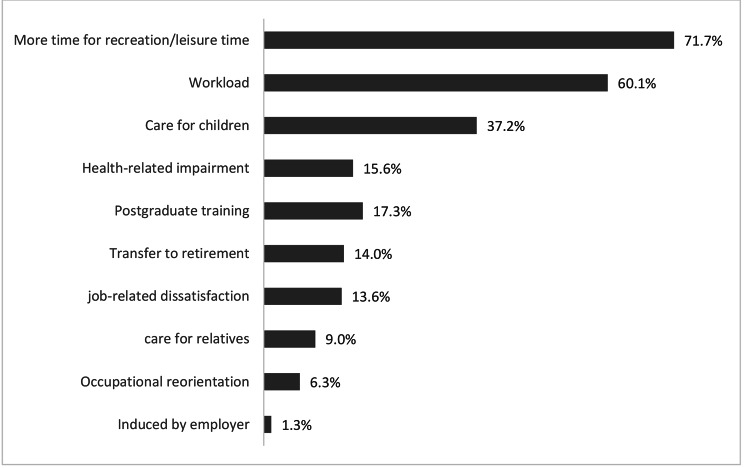
Reasons for wanting to reduce work hours (in %)

In addition to socio-demographic and job-specific aspects (e.g. working hours, institution, type of employment), questions about plans regarding work hour preference were integrated. First, physicians were asked whether they want to change their work hour (reduce or increase working hours, do not want to change). In addition, those you stated that they prefer to reduce work hours, were asked to give reasons (see Fig. 1, allowing for multiple answers). Using the Utrecht Work Engagement scale [27, 33], perception of work was analyzed using nine items and a seven-point-scale. This scale is commonly used to measure engagement within health care settings.

Burnout was investigated using the Copenhagen Burnout Inventory [4], that consists of 19 items and a five-point scale. The scale was adapted for professionals working in health care [6] and can be structured into three subscales: personal burnout (e.g. “How often do you feel tired?”, 100 = always/ 0 = never), work-related burnout (e.g. “Is your work emotionally exhausting?, 100 = to a very high degree/ 0 = to a very low degree) and patient-related burnout (e.g. “Are you tired of working with patients?”, 100 = to a very high degree/ 0 = to a very low degree).

### Data Analysis

Data was analyzed using STATA 16 SE statistical software. Apart from descriptive evaluations (independent t-tests for continuous variables and chi^2^-tests for categorical variables), regression models were part of the statistical analysis. In addition, causal mediation analysis was conducted using the *medeff* package [34], in order to detect direct, indirect and overall effects. Mediation models examined these effects using the three dimensions of burnout as independent variables, work engagement as mediating variable and work hours reduction as the outcome variable. Adjustments were made for age, gender, presence of children and marital status. Effect sizes were calculated using Cohen’s d (small effect: d = 0.2; medium effect: d = 0.5; large effect: d = 0.8) as well as Cramer’s V (small effect: V = 0.1; medium effect: V = 0.3; large effect: V = 0.5).

Cases with missing values with regard to the survey instruments were not included in the final analyzes (percentage of missing values: <4%, [35]). A significance level of 0.05 was assumed for all statistical evaluations.

## Results

Overall, 725 physicians with a variety of medical specialities could be included into analysis. The overall sample was divided into two sub-groups (‘wanting to reduce working hours’ and ‘not wanting to reduce working hours/wanting to increase working hours’), as only 15 subjects (2% of the overall sample) want to work more. Sociodemographic details on the sub-groups based on their decision to reduce working hours or not, are summarized in Table 1.

**Table 1:** Descriptive information with regard to desired changes in work hours

	Reduction of working hours (n = 297)	No reduction of working hours (n = 428)	p-value
Age	Ø 44.9	Ø 43.6	n.s.
Gender			
female	40.7%	37.6%	n.s.
male	59.3%	62.4%	
Care work for children (“yes”)	75.8%	74.8%	n.s.
Marital status			
married	64.0%	60.5%	n.s.
in a relationship	22.6%	21.3%	
single	13.5%	18.2%	
Working hours (including on-call duty and overwork)	Ø 52.6	Ø 46.5	p < 0.001, d = -0.529
Leading position („yes“)^1^	25.0%	24.9%	n.s.
Medical setting			
outpatient	35.4%	34.8%	n.s.
inpatient	64.6%	65.2%	
Burnout^3^			
Personal	50.8	42.0	p < 0.001, d = -0.474
Patient-related	26.4	21.3	p < 0.001, d = -0.278
Work-related	40.9	34.6	p < 0.001, d = -0.440
Utrecht Work Engagement Scale^4^	3.5	3.8	p < 0.001, d = 0.258
Satisfaction with working time schedule			
Satisfied	31.6%	85.5	p < 0.001, V = 0.550
Dissatisfied	68.4%	14.5%	

Note: ^1^= senior or chief physician; n.s. = not significant; ^3^= range: 0-100, higher score means greater burnout; ^4^= possible range: 0.33-6.0, higher score means greater engagement; n.s. = not significant; d = Cohen’s d effect size; V = Cramer’s V effect size.

Overall, 297 physicians (women: 40.7%; men: 59.3%) wanted to reduce their work hours compared to 428 physicians (women: 37.6%; men: 62.4%) that were not planning to work less. Physicians that aimed to reduce their current work hours stated a reduction of 11.5 h on average. About 85% of this group were not satisfied with their current working time. Contrary, in the group of physicians that do not want to work less, 14.8% were not satisfied with their working time. Reasons for reduction of work hours are summarized in Fig. 1. According to the data set, “having more time for leisure and recreation” as well as due to the “workload” were most often stated as reasons why physicians in this sample aimed to reduce their work hours.

Additionally, regression models were applied to further analyse the association between work hours reduction and burnout as well as work engagement (Table 2), controlling for sociodemographic characteristics (gender, age, having children and marital status).

**Table 2:** Multiple regression analyses with “Work hours reduction” as a predictor of the three dimensions of burnout and work engagement

	Patient-related Burnout	Personal Burnout	Work-related Burnout	Work Engagement
Work hours (no reduction^a^)	5.05***	9.31***	6.69***	-1.39**
Age	0.02	-0.27***	-0.04	0.03
Gender (male^b^)	-0.54	5.20***	4.15***	-0.07
Children (yes^c^)	5.33**	2.79	4.78**	-1.32*
Marital status (married^b^)				
in a relationship	0.69	-1.99	-2.04	0.01
single	-0.75	-0.79	-0.56	0.99
constant	19.42	50.57	33.22	21.44
R^2^	0.03	0.11	0.09	0.04

Note: UWES = Utrecht Work Engagement Scale; ^a^reference category coded as “0” = no reduction in working hours, “wanting to reduce” was coded as “1”; ^b^reference category coded as “0” = male subjects; ^c^reference category coded as “0” = having children; ^d^reference category coded as “0” = married;*p ≤ 0.05; **p ≤ 0.01; ***p ≤ 0.001.

The regression analyses - focussing on all three dimensions of burnout as the outcome variable - show that physicians who aimed to reduce their working hours exhibited significantly more burnout with regard to all three constructs. In addition, female subjects exhibited more personal, as well as work-related burnout and being younger was related to greater levels of personal burnout. Moreover, not having children was significantly linked to higher levels of both patient- and work-related burnout.

Furthermore, lower levels of work engagement were associated with wanting to cut down work hours. With regard to sociodemographic information, “having no children” was associated with lower levels of work engagement. In all regression models, marital status was neither significantly linked to burnout nor work engagement.

**Fig. 2:**
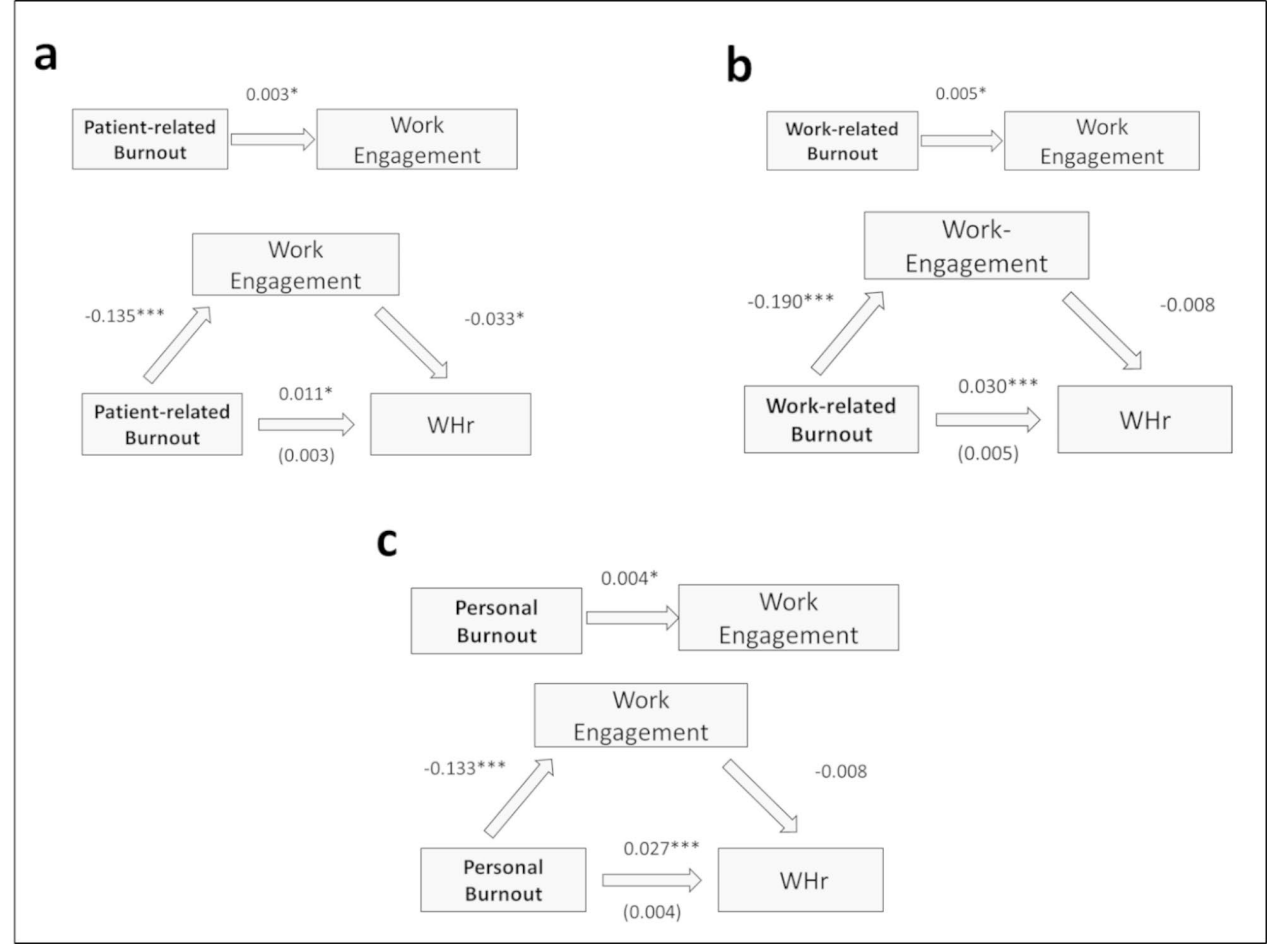
Causal mediation analysis with work engagement as the mediator between burnout and work hour changes Note: a = mediation between patient-related burnout and change in work hours; b = mediation between work-related burnout and change in work hours; c = mediation between personal burnout and change in work hours; WHr = work hours (reduction vs. no change).

Causal mediation analysis was performed (described in Fig. 2) to investigate whether the relationship between burnout and the desire to reduce working hours may be mediated by work engagement. For all three dimensions of burnout, significant direct effects of burnout on the desire to work less as well as significant mediated effects of burnout through work engagement were found (Fig. 2). For work engagement, significant direct effects of work load on working hour reduction were observed (patient-related: b = 0.003, p < 0.05; work-related: b = 0.005, p < 0.05, personal: b = 0.004, p < 0.05 ). Moreover, significant mediation effects of burnout through work engagement on working hour reduction could also be observed (patient-related: b = − 0.135, p < 0.001; work-related: b = − 0.190, p < 0.001; personal: b = − 0.133, p < 0.001 ). However, only for patient-related burnout the percentage of total effect through mediation was reasonable high (patient-related: 29.1%; work-related: 5.0% personal: 3.8%).

## Discussion

The aim of the study was to investigate work hour preferences with regard to burnout and work engagement in a sample of physicians working in different specialties. The impact of burnout on the clinical population as well as on patient safety and quality of care is widely acknowledged and more and more physicians prefer to reduce their work hours in order to reduce burnout [36–39].

Overall, approximately 41% of physicians in this sample considered to reduce their work hours. In addition, 14.8% of physicians that do not want to work less at the time of the data collection, were dissatisfied with their work hours. According to preliminary studies, 29–49% of employees working in health care planned to reduce work hours or work part time [30, 40, 41]. With regard to our findings, none of the sociodemographic factors revealed any significant differences with regard to work hour changes. In other words, the desire to work less did not depend on gender, age or the presence of children (living in the same household). Other factors may be more relevant in this context. Therefore, future studies may focus on the relevance of the children’s age such as differences in intensity of care being necessary or the partner’s employment situation if the physician is in a relationship.

Apart from shortage of health care professionals, to the medical profession itself may often be accompanied by increases in workload and burnout, which has also been shown to be a reason for physician’s decision to resign from medical profession or work part time [14, 42]. Especially younger physicians may see part time work as a solution to reduce heavy workload, being able to find the balance between work and private life [38]. This is also reflected in the current study. According to the current data, 60.1% of physicians stated “workload” and 71.7% stated “leisure time” as the main reasons for work hour reduction. Previous studies suggest, that having children would increase the overall burden, leading to work-home-conflicts that also influence career decisions of physicians [42]. In the current study, 37.2% mentioned child care as a reason for work hour reduction. In addition, age has been related to lower levels of psychological distress and burnout in physicians [43, 44]. So far, evidence regarding different levels of burnout exhibited by different medical specialties or between physicians working in hospitals or ambulatory settings is rather mixed. Regression analyses in our study (including age, gender, having children and marital status as control variables) revealed, that the desire to reduce work hours was linked to higher burnout on all three dimensions. In other words, the desire to work less was associated with higher levels of patient-related, work-related and personal burnout in a broad sample of physicians with different sociodemographic and occupational characteristics (such as medical settings and specialties).

In order to reduce burnout and therefore maintain the medical work force by preventing physicians to cut down their work hours, previous studies concentrated on work-related parameters that may help to reduce work-related stress within the medical profession. In this context, there is evidence, that work engagement and work motivation may be positively linked to (personal) burnout [45]. In our study, the level of work engagement significantly differed between physicians that want to reduce work hours and physicians that do not plan to work less hours In other words, physicians with the desire to work less were characterized by lower levels of work engagement. The question remains whether interventions that aim to increase work engagement and commitment, may also be helpful to keep physicians’ work force on a sufficient level (i.e. no reduction in work hours). Improving work engagement may help to improve emotional and physical health, as well as coping with job-related stress. This in turn could reduce the risk for developing burnout [46].

In order to further investigate whether work engagement may be linked to the relationship between burnout and the desire to work less, mediation analyses revealed the following results. Significant effects were found with regard to work engagement. Physicians with high levels of burnout (patient-related, work-related and personal) expressed less work engagement and were more likely to plan a reduction of their working time. Since a shortage of physicians has been observed in many medical professions worldwide, this may increase physician shortage even more and result in further increase of workload exhibited by their colleagues. Therefore, our findings have important implications for all stakeholders working in healthcare settings. The concept of work engagement may be helpful in this context and should be enhanced by supervisors or health care institutions. Additionally, it has been suggested that interventions, that focus on reducing job demands (i.e. having sufficient time for work) and strength resources instead have the greatest potential to improve physicians’ well-being as well as patient care [28, 47]. In this context, efforts at enhancing work engagement may help to mitigate burnout risk [46]. Retaining the medical work force and maintaining delivery of high standard patient care is important to face future challenges of healthcare systems worldwide.

### Limitation

Results of the work engagement scale showed, that the overall scores were lower compared to other studies investigating physicians [48, 49] but reflect findings from a similar study investigating burnout, work engagement and patient care [28]. Even though, mediation analysis revealed significant direct as well as mediation effects, the total effects with regard to of work-related and personal burnout were relatively low, which should be kept in mind when interpreting these results.

The participating physicians in this sample were asked for their medical specialties, however, due to multiple answers, it was not possible to conduct stratified analyses to further investigate possible differences between physicians. In addition, due to the study’s cross-sectional design, questions in terms of causality cannot be answered. Future research is required to expand these analyses and help to design and establish occupational interventions that prevent burnout, increase work engagement and keep physicians within health care systems. In this context, prospective research should investigate whether different working hour arrangements – such as working from home or offering telemedical services – may be useful in decreasing the negative consequences associated with burnout and help to anticipate physician shortage by enhancing self-efficacy and resilience.

## Data Availability

The data set and materials supporting the results are available from the corresponding author upon reasonable request.
